# A co-alteration parceling of the cingulate cortex

**DOI:** 10.1007/s00429-022-02473-2

**Published:** 2022-03-03

**Authors:** Jordi Manuello, Lorenzo Mancuso, Donato Liloia, Franco Cauda, Sergio Duca, Tommaso Costa

**Affiliations:** 1 GCS fMRI, Koelliker Hospital and University of Turin, Turin, Italy; 2 FOCUS Lab, Department of Psychology, University of Turin, Turin, Italy; 3 Neuroscience Institute of Turin, Turin, Italy

**Keywords:** Cingulate cortex, Retrosplenial cortex, Bayesian statistic, Morphometric co-alteration network, Hierarchical clustering

## Abstract

The cingulate cortex is known to be a complex structure, involved in several cognitive and emotional functions, as well as being altered by a variety of brain disorders. This heterogeneity is reflected in the multiple parceling models proposed in the literature. At the present, sub-regions of the cingulate cortex had been identified taking into account functional and structural connectivity, as well as cytological and electrochemical properties. In the present work, we propose an innovative node-wise parceling approach based on meta-analytic Bayesian co-alteration. To this aim, 193 case–control voxel-based morphometry experiments were analyzed, and the Patel’s *κ* index was used to assess probability of morphometric co-alteration between nodes placed in the cingulate cortex and in the rest of the brain. Hierarchical clustering was then applied to identify nodes in the cingulate cortex exhibiting a similar pattern of whole-brain co-alteration. The obtained dendrogram highlighted a robust fronto-parietal cluster compatible with the default mode network, and being supported by the interplay between the retrosplenial cortex and the anterior and posterior cingulate cortex, rarely described in the literature. This ensemble was further confirmed by the analysis of functional patterns. Leveraging on co-alteration to investigate cortical organization could, therefore, allow to combine multimodal information, resolving conflicting results sometimes coming from the separate use of singular modalities. Crucially, this provides a valuable way to understand the pathological brain using data driven, whole-brain informed and context-specific evidence in a way not yet explored in the field.

**Supplementary Information:**

The online version contains supplementary material available at 10.1007/s00429-022-02473-2.

## Introduction

The cingulate cortex has captured the attention of brain academics since the early work of Broca (1878). A few decades afterwards, Brodmann introduced the idea that the cingulate cortex was not a unitary cytoarchitectonic structure, proposing the subdivision into precingulate (BAs 24, 25, 32, 33; mainly agranular) and postcingulate (BAs 23, 31; granular) sub-regions (Brodmann 1909). The heterogeneity of the cingulate cortex was further supported by the advent of functional MRI, showing its involvement in several brain functions and cognitive domains, as emotion (Rolls 2019), decision-making (Lockwood and Wittmann 2018), motor behavior (Caruana et al. 2018), pain (Benarroch 2020), consciousness (Manuello et al. 2016), and memory (Maguire 2001). The analysis of resting state generally associated the anterior cingulate cortex with the salience network (Seeley et al. 2007), and the posterior cingulate with the default mode network (DMN) (Andrews-Hanna et al. 2010; Buckner et al. 2008; Cauda et al. 2010; Fan et al. 2019).

This varied functional fingerprint suggested the existence of even more than two parcels in the cingulate cortex, finding further support by anatomical and cytological evidence (Caruana et al. 2018; Jin et al. 2018; Palomero-Gallagher et al. 2009; Vogt and Vogt 2003). Similarly to many other brain structures, there is currently no general consensus on the most appropriate number of parcels to be used to analyze the cingulate cortex. This level of granularity varies also depending on the modality under investigation. By now, the connectivity-based identification of structures and sub-structures has been based mainly on the analysis of structural or functional properties, or a combination of the two (Albers et al. 2021; Zalesky et al. 2010). However, the results of a recent work by Nani et al. (2021) suggest that a further approach could come from the analysis of structural co-alteration. This is particularly relevant if considering that the choice of a best atlas, for example for node definition in network analysis or a priori ROI design, mainly depends on the specific research question (Messé 2020). What is therefore the better fit when investigating the pathological brain? Although the relation between functional and structural normative connectivity and pathological networking has been highlighted (Cauda et al. 2018; Vanasse et al. 2021), it cannot be ruled out the possibility that the adoption of a not modality-specific parceling schema could result in the loss of fine details and consequent imprecise results. Nani et al. (2021) opened the way showing that different patterns of co-alteration can be observed for sub-regions of a same brain structure, with some of them violating what would be expected on the basis of healthy functioning. However, those analyses were based on pre-existing parceling of the insular cortex informed by the healthy brain only (Kelly et al. 2012). Here we aimed to build a second pillar proposing a data-driven approach to identify sub-regions on the basis of features of the pathological brain. An advantage of such conceptualization could be its ability to synthesize sometimes diverging levels of analysis into a unique measure of brain similarity having clinical relevance, combining, and extending, functional and structural information. Moreover, this kind of approach can help to deepen the understanding of the involvement of specific brain regions in neurodegenerative and psychiatric disorders. Notably, the use of statistical metrics based on co-alteration was found to outperform traditional methods based on localization in the identification of core regions of specific pathologies, suggesting that the phenomenon of co-alteration captures meaningful properties of the brain (Cauda et al. 2020). The need for such form of advancement would be particularly relevant in the case of cingulate cortex. In fact, although in last years it was described as involved in several pathologies, such as Parkinson’s disease (Vogt 2019b), mild cognitive impairment (Sambuchi et al. 2019), ADHD (Vogt 2019c), schizophrenia (Liloia et al. 2021a), autism spectrum disorder (Lukito et al. 2020), Alzheimer’s disease (Mutlu et al. 2016), and PTSD (Hinojosa et al. 2019), it has been pointed out that the comprehensive role of cingulate cortex in neuropathology is still elusive (Vogt 2019a). Previous evidence based on a cross-disorder approach showed that the cingulate cortex is involved in a high amount of disorders (Cauda et al. 2019; Goodkind et al. 2015; Liloia et al. 2018), and its structural alteration is reported with higher frequency than other brain regions (Nani et al. 2021). However, no details were available on the possible differential role of its sub-regions. With this in mind, the adoption of a cross-disorder perspective could enable the investigation of general pathological processes shared among different diseases (Buckholtz and Meyer-Lindenberg 2012). In the present work, we aimed to understand if the spatial information concerning the relationship between the cingulate cortex and the rest of the brain in clinical conditions (defined as co-alteration network) could highlight an intrinsic organization of the cingulate-cortex itself. Hierarchical clustering was used to identify, in a data-driven manner, sub-regions with a distinguishable co-alteration profile. Clustering is not new in the topic of parceling the cingulate cortex. In past years, Palomero-Gallagher et al. (2009) used this approach on the profile of receptors binding to better characterize Brodmann’s bipartite and Vogt’s four-region neurobiological models. Using task-fMRI meta-analytic data instead, Torta et al. (2013) distinguished 3 clusters. More recently, Jin et al. (2018) identified 6 functional and 10 anatomical sub-regions applying *K*-means clustering to resting state and DTI data, respectively. What distinguishes the present work is therefore the leveraging on the co-alteration information, which, to the best of our knowledge, had never been considered before. The comparison of our results with the other existing models could help to elucidate functional, anatomical, and biochemical properties of the cingulate-cortex. Moreover, co-alteration based sub-regions could then be used as domain-specific a priori for further research on brain disorders.

## Materials and methods

### Selection of studies

Experiments were retrieved from the voxel-based morphometry (VBM) section of BrainMap (Fox et al. 2005; Fox and Lancaster, 2002; Laird et al. 2005) using the software *Sleuth v.3.0.4*. BrainMap is among the largest existing international repositories of neuroimaging data (Vanasse et al. 2018) comprising, at the moment of the search (March 2021), 1002 VBM articles, for a total of 3179 experiments, 81,496 subjects, and 22,332 locations. To assess the extent of structural alteration on the cingulate cortex, we performed the following query:

[Experiments Contrast is Gray Matter] AND [Experiment Context is Disease] AND [Observed Changes is Controls > Patients] AND [Locations MNI image is cingulate_cortex_mask].

This search allowed to retrieve VBM experiments reporting at least one focus of structural alteration in the cingulate cortex, often along with further spots of alteration in different brain regions. Specifically, we decided to focus on the decrease contrast only, which is generally considered a marker of atrophy of GM (Nani et al. 2021) (see Supplementary methods and Fig. S1 for details of the literature search process). The binary mask of the cingulate cortex (6308 voxels, 2 mm resolution) used in the search was obtained combining the following 10 parcells of the AAL atlas (version 3.1) (Rolls et al. 2020): Cingulate_Mid_L, Cingulate_Mid_R, Cingulate_Post_L, Cingulate_Post_R, ACC_sub_L, ACC_sub_R, ACC_pre_L, ACC_pre_R, ACC_sup_L, ACC_sup_R (see Fig. S2).

### Estimating spatial convergence among selected experiments

Being in a meta-analytic framework, it is necessary to assess the level of coherence between the experiments previously selected. In this case, the VBM data included in the pool after screening were statistically elaborated using the anatomical likelihood estimation (ALE) algorithm, a quantitative method allowing to estimate consistent morphological alterations across a set of neuroimaging studies (Eickhoff et al. 2012, 2009; Turkeltaub et al. 2012). Being a technique to run coordinate-based meta-analysis, ALE is based on the analysis of the *x*–*y*–*z* coordinates of the peaks of effect (i.e. foci) as made available in the original publications. A Gaussian probability distribution is built around each focus to model the original alteration it represents:

$$p\left( d \right) = \frac{1}{{\sigma^{3} \sqrt {\left( {2\pi } \right)^{3} } }}e^{{ - \frac{{d^{2} }}{{2\sigma^{2} }}}} ,$$

where *d* is the Euclidean distance between voxels and the considered focus, while *e* models the spatial uncertainty. $\sigma$ is instead expressed as

$$\sigma = \frac{{{\text{FWHM}}}}{{\sqrt {8\ln 2} }},$$

where FWHM is the full-width half-maximum.

In this way, a modeled activation (MA) map is generated for each experiment. Notably, the size of the Gaussian kernel varies between the MA maps, allowing to model the spatial uncertainty due to the differences in sample size used in each included experiment (Eickhoff et al. 2016). The union of the MA maps constitutes the result of the ALE, where the value of each voxel is a measure of the likelihood of its alteration given the specific set of experiments analysed. Therefore, the ALE map represents the whole brain distribution of GM co-alterations with the cingulate cortex, that is a pattern of altered voxels which includes different brain regions along with the cingulate cortex.

### Construction of the structural co-alteration network

To estimate dependencies between the cingulate cortex and the other brain co-altered regions, the morphometric co-alteration network (MCN) of the cingulate cortex was built, following the methodology described in Manuello et al. (2018). This kind of analysis can determine the existence of a statistical relationship between the alteration of the cingulate cortex and the co-occurring alteration of other brain areas. In the obtained network, nodes represent regions found to be altered in the experiments retained in the pool, whereas edges link couples of nodes for which co-alteration is more likely than independent alterations. To determine the nodes, the previously obtained ALE map was fed to a peak detection algorithm. This data-driven method allowed to place them where our dataset indicated a large confluence of GM alterations. Dependencies between the nodes were then computed using the Patel’s *κ* Bayesian index (Patel et al. 2006). Let us consider the couple of nodes A and B, and their state in the first experiment. If the coordinates of A point to a region showing non-zero value in the associated MA map, node A is considered altered in that given experiment. The same applies to node B. Therefore, in the first experiment four cases exist: both A and B are altered; A is altered and B is not altered; B is altered and A is not altered; none of the two is altered. Once the state of A and B had been verified for each experiment in the dataset, the following four probabilities can be computed:

$$\begin{aligned} \theta_{1} & = P\left( {a = 1,b = 1} \right) \\ \theta_{2} & = P\left( {a = 1,b = 0} \right) \\ \theta_{3} & = P\left( {a = 0,b = 1} \right) \\ \theta_{4} & = P\left( {a = 0,b = 0} \right), \\ \end{aligned}$$

where 0/1 codes the state of the node (i.e. unaltered/altered). For each couple of nodes, the marginal probabilities can be hence computed as reported in Table 1.

**Table 1:** Marginal probabilities between altered and unaltered nodes

	Node a		
	Altered	Unaltered	
Node b			
Altered	$\theta_{1}$	$\theta_{3}$	$\theta_{1} + \theta_{3}$
Unaltered	$\theta_{2}$	$\theta_{4}$	$\theta_{2} + \theta_{4}$
	$\theta_{1} + \theta_{2}$	$\theta_{3} + \theta_{4}$	1

On the grounds of these probabilities, the Patel’s κ was computed as follows:

$$\kappa = \frac{{\theta_{1} - E}}{{D\left( {\max \left( {\theta_{1} { }} \right){-}E} \right) + \left( {1 - D} \right)\left( {E - \min \left( {\theta_{1} } \right)} \right)}},$$

where

$$\begin{aligned} E & = \left( {\theta_{1} + \theta_{2} } \right)\left( {\theta_{1} + \theta_{3} } \right) \\ \max \left( {\theta_{1} } \right) & = {\text{min}}\left( {\theta_{1} + \theta_{2} ,\theta_{1} + \theta_{3} } \right) \\ \min \left( {\theta_{1} } \right) & = {\text{max}}\left( {0,2\theta_{1} + \theta_{2} + \theta_{3} - 1} \right), \\ \end{aligned}$$

and

$$D = \left\{ {\begin{array}{*{20}c} {\frac{{\theta_{1} - E}}{{2\left( {\max \left( {\theta_{1} } \right) - E} \right)}} + 0.5 \quad {\text{if}} \theta_{1} \ge E} \\ {0.5 - \frac{{\theta_{1} - E}}{{2\left( {E - \min \left( {\theta_{1} } \right)} \right)}}\quad {\text{otherwise}}{.}} \\ \end{array} } \right.$$

This index can, therefore, measure the probability of co-alteration of a and b against the probability of their independent alteration. Edges with a Patel’s *κ* value close to 1, that is, the upper bound of the continuous range of values obtainable through this index, indicate a high likelihood of co-alteration of the two nodes linked by it.

Once the complete whole brain network was obtained, the nodes anatomically located inside the cingulate cortex mask previously used during the data search were identified and considered as root nodes. The profile of connectivity of each root node with its first neighbors was retained for the following analyses.

### Hierarchical clustering of the root nodes

**Fig. 1:**
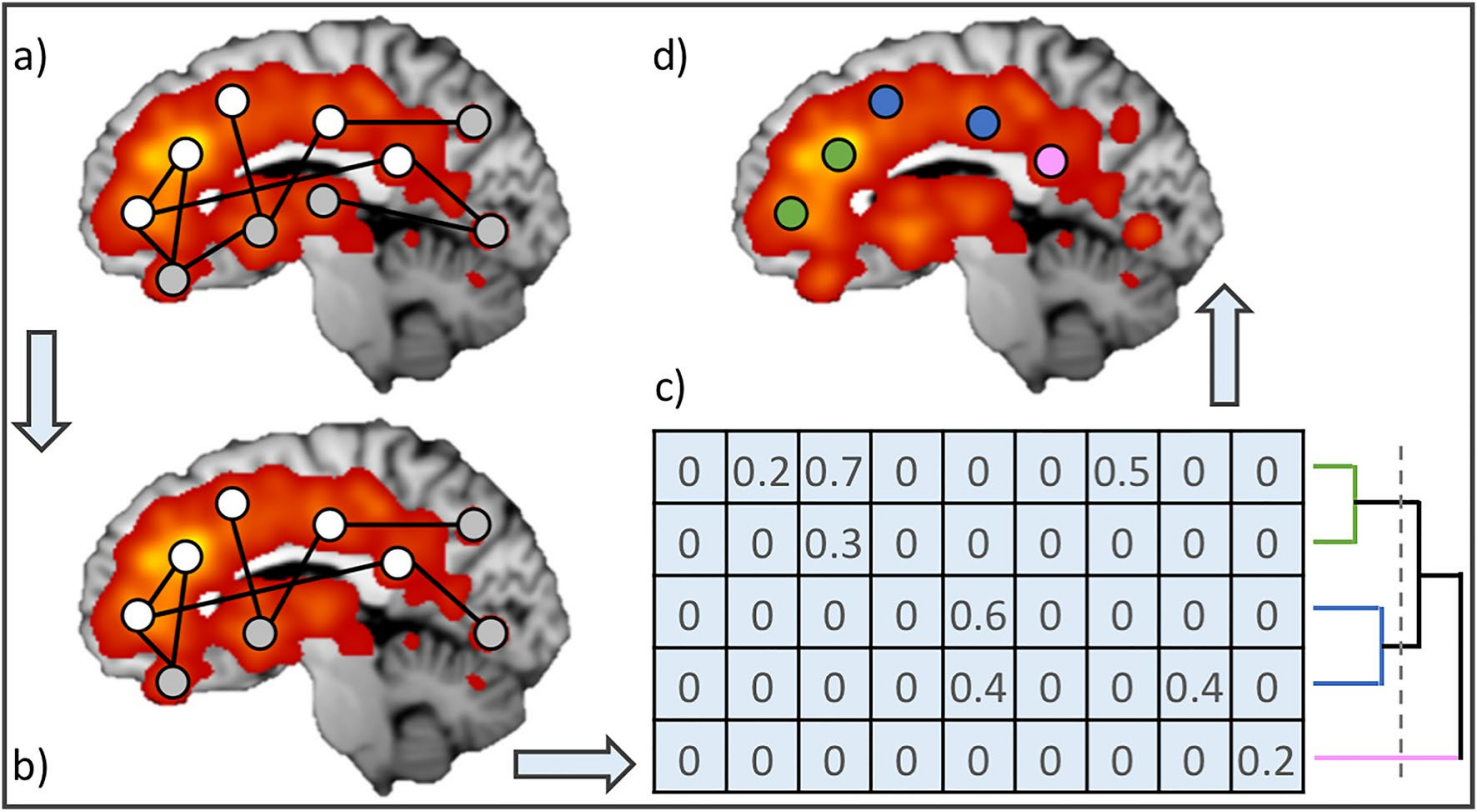
Graphical representation of the pipeline used. **a** The MCN is obtained on the basis of the ALE map. Root nodes (i.e. localized in the cingulate cortex) are colored in white, while nodes in the rest of the brain are colored in gray; **b** only first neighbors of the root nodes are retained, and edges between couples of non-root nodes are removed; **c** the root nodes × first neighbor nodes matrix is built. Values are based on the Patel’s *κ*, representing the likelihood of co-alteration between each couple of nodes. This matrix is used as input for the hierarchical clustering of the root nodes (i.e. computed between rows); **d** clustering results are visualized on the brain. In this case, colours refers to *c* = 3, as marked with the dashed line on the dendrogram in (**c**). For the sake of clarity, the visualization is based on synthetic and simplified data rather than on the real one used for the analyses

The MCN is usually the end product of this kind of analyses. Conversely, in the present study, the previously obtained connectivity profiles of the root nodes were combined to build a matrix in which rows represent the root nodes, and columns represent all the nodes of the whole brain co-alteration network. Based on this input, hierarchical clustering was performed using Pearson correlation as similarity metric (computed between rows) and Weighted Pair Group Method with Arithmetic Mean (WPGMA) (Sokal et al. 1958) as linkage method (see Fig. 1 for a graphical representation of the complete pipeline). One of the advantages of this clustering approach is that it allows to examine results on multiple scales (Kelly et al. 2012), which is particularly relevant in the case of unknown preferable number of sub-parcels in a brain region (Cauda and Vercelli, 2013). Moreover, results are based on whole-brain properties, taking into account the multivariate relationship between multiple nodes at the same time. To test possible effects of the linkage method used, average and complete linkage were also alternatively used. Clustering procedure was implemented in Orange v.3.3.7 (Demsar et al. 2013). The obtained dendrogram, therefore, describes the grouping of the root nodes based on their profile of structural co-alteration with the rest of the brain.

Although the clustering procedure was implemented at node-wise level (i.e. the input was a nodes × nodes matrix), we also generated a set of voxel-wise maps, one for each cardinality of the clustering solution. To do so, each voxel in the ROI of the cingulate cortex was assigned to the same cluster of its nearest root-node, on the basis of Euclidean distance. The voxel-wise parceling can be downloaded from figshare (10.6084/m9.figshare.16708816.v1) (See also Fig. S6).

The analyses described so far mainly concerned the relationship between the cingulate cortex and the rest-of the brain. However, local co-alteration between different sections of the cingulate cortex itself (i.e. within-cingulate cortex) may have a role in shaping the whole brain MCN (Cauda et al. 2020; Zamani Esfahlani et al. 2020). In order to evaluate if local co-alteration was driving the observed effect, the node-wise clustering procedure was repeated after discarding the edges among root nodes. Complementary, we verified whether or not local co-alteration was coherent with the identified parceling of the cingulate cortex based on the whole brain information.

### Decomposition into functional networks

Since the previous analyses highlighted a cluster of fronto-parietal nodes compatible with the DMN, a functional network decomposition was implemented to further verify if, although based on structural data, the possible involvement of the DMN was supported by functional evidence as well. Specifically, the first-neighbors of the root nodes composing the cluster of interest cutting the dendrogram at *c* = 2 were identified on the basis of the co-alteration edges. Then, the number of nodes located in each of the 7 resting state networks, as they are defined in the work of Yeo et al. (2011), was counted. This step was further iterated thresholding the root-to-first-neighbors network to the 30th, 60th and 90th percentile of the Patel’s *κ* values distribution.

### Multi-dimensional scaling of the root nodes

To further describe the relationships among the root nodes, the same similarity matrix previously computed (i.e. whole-brain and local co-alteration) was used as input for multi-dimensional scaling (MDS). The two-dimensional MDS was initialized with principal coordinate axes approach (Torgerson 1958) as implemented in Orange v.3.3.7 (Demsar et al. 2013). Of note, the aim of the MDS technique is not to create clusters, but rather to produce a spatial representation of a set of elements (i.e. the root nodes) in a n-dimensional space (i.e. 2D in this case). However, it is possible to verify the convergence between the MDS solution and the hierarchical clustering dendrogram. If nodes belonging to different clusters are well segregated in the MDS solution, this means that MDS and hierarchical clustering are coherent one to each other.

### Relationship with functional connectivity

All the analyses described above were based on structural data of alteration. However, to better characterize the co-alteration based parceling, we also evaluated the possible relationship with functional resting state connectivity. To do so, the coordinates of each root node were used to obtain a whole brain seed-voxel correlation (SVC) map from the Neurosynth portal (Yarkoni et al. 2011). These maps were based on the resting state data of 1000 healthy subjects, processed as described in Yeo et al. (2011). The SVC maps were then grouped based on the previously obtained clustering results (i.e. based on whole-brain co-alteration). For each level of cardinality, the average Pearson correlation among within-cluster and between-cluster SVC maps was computed and compared. Within-cluster values higher than between-cluster values would suggest coherence between structural co-alteration and functional connectivity. Finally, as an additional analysis, Orange was used to perform hierarchical clustering of the SVC maps, choosing Pearson correlation and WPGMA as in the case of the co-alteration based clustering.

## Results

### The co-alteration network of the cingulate cortex

After screening and selection steps, 194 VBM experiments remained in the pool, for a total of 2985 foci of alteration, and 4941 clinical subjects (see also Figure S1 and Table S1). The obtained whole brain MCN counts 857 nodes. 30 of them were localized in the cingulate cortex (21 in the left hemisphere). The sub-network limited to the first neighbors of the root nodes consisted of 418 nodes covering the whole brain, and 1861 undirected edges (141 edges among root nodes). All the root nodes were linked to at least one node outside the cingulate cortex.

### Co-alteration-based clustering and MDS

**Fig. 2:**
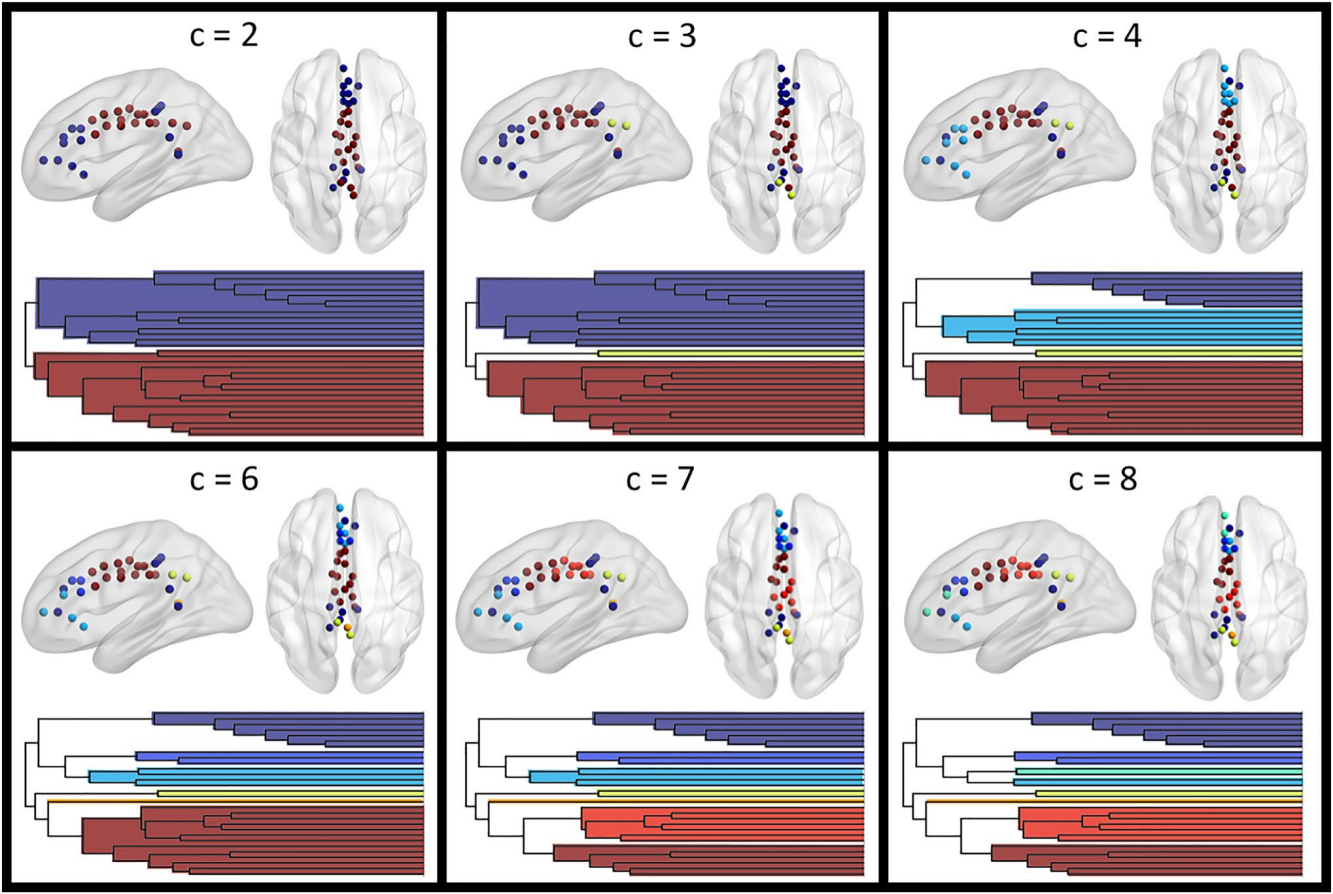
Results of the hierarchical clustering of the 30 root nodes, obtained using Pearson correlation and WPGMA. Axial views are in neurological convention (left is left). *C* = 5 is not shown since only the orange posterior node changed with respect to *c* = 4. Colors are coherent between the dendrogram and the visualization of the nodes

**Fig. 3:**
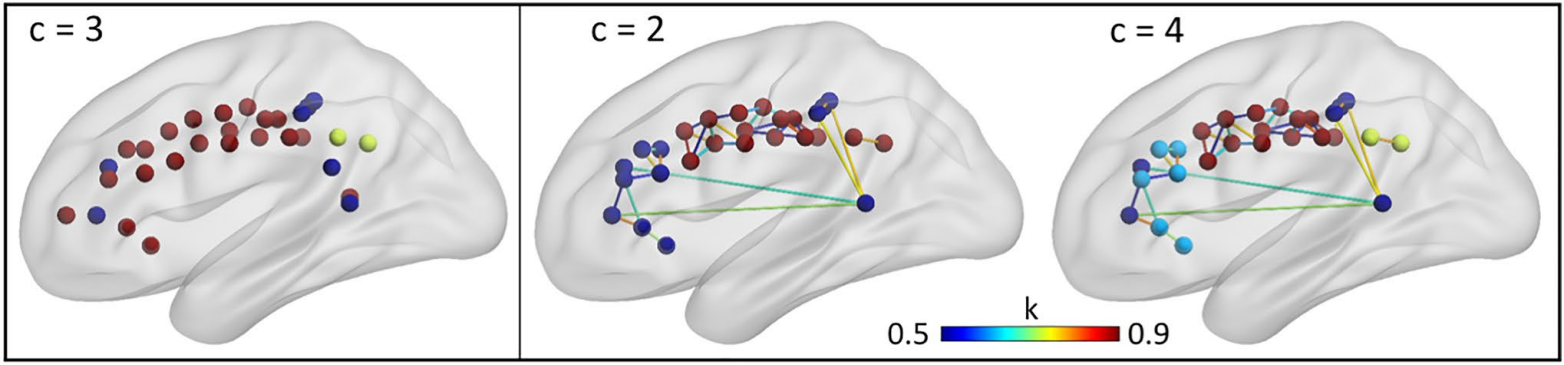
Evaluation of the role of local co-alteration. Left: hierarchical clustering result at *c* = 3, obtained after excluding edges between root nodes in the cingulate-cortex. Right: details of local co-alteration between root nodes. Edges’ color from blue to red represents increasing Patel’s *κ* values (thresholded at 0.5 for visualization purpose). Nodes’ colors are based on the clusters obtained for the whole MCN (including both local and global co-alteration), and hence it is coherent with Fig. 1. Nodes with no edges surviving the imposed κ threshold were not shown

The hierarchical clustering was therefore applied to a 30 × 418 matrix (root nodes × first neighbor nodes), containing Patel’s *κ* values for 1861 undirected edges. The bipartite solution (corresponding to the highest branch of the dendrogram) highlighted an interesting cluster including nodes in both the rostral and the caudal portion of the cingulate cortex (Fig. 2). Notably, the core of this fronto-parietal ensemble survived up to the solution with 20 clusters (Fig. S3). Indeed, five of these nodes are the first to be clustered in the dendrogram, suggesting a very high similarity between their profile of co-alteration (Fig. S4). This fronto-parietal trend was confirmed also when using either average linkage or complete linkage (Fig. S5). Coherently, it was still present after removing the effect of within-cingulate cortex co-alteration, and further highlighted by the analysis of co-alteration within root nodes (Fig. 3). Overall, the local component of the MCN was particularly consistent with the repartition of the cingulate cortex into 4 sub-regions. It is interestingly to note that local co-alteration highlights the role of the single node in the left retrosplenial cortex, linking other two groups of nodes in the fronto-parietal ensemble not showing direct co-alteration between them with a value of *κ* ≥ 0.5. Notably, those 4 edges appear to cover a longer range than the other local ones, proceeding across spatially close and contiguous nodes. The within-cingulate cortex co-alteration also confirmed the segregation of the two most caudal nodes in the posterior cingulate cortex, that despite the physical proximity are neither linked with the caudal section of the fronto-parietal cluster, nor with the other nodes in the middle/posterior cingulate cortex. Coherently with what observed for the fronto-parietal ensemble, those two nodes are still a well-segregated cluster even after removing local co-alteration, as well as if considering local and global components together. In this case, the small cluster is already present at *c* = 8, and persists up to *c* = 3. Descending along the dendrogram it is possible to observe a higher heterogeneity in the anterior cingulate cortex, compared with the middle cingulate cortex (Fig. 2), as suggested by the presence in *c* = 8 of clusters with higher cardinality in the middle portion (See Fig. S6 for the voxel-wise version of the parceling). Overall, the clustering exhibits symmetry between the hemispheres.

**Fig. 4:**
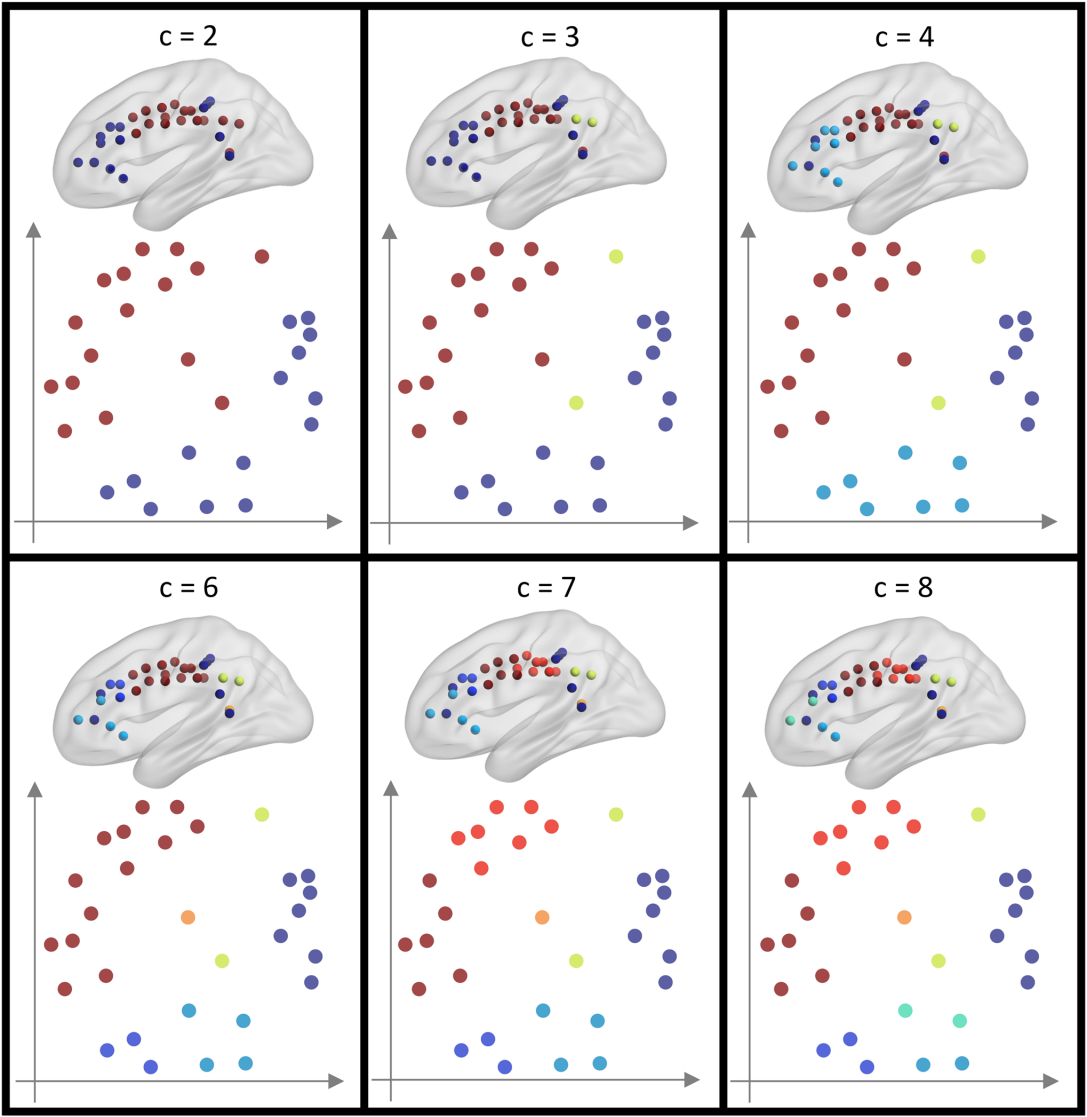
Results of the MDS. Nodes in the obtained 2D spatial distribution were colored based on the results of hierarchical clustering at different cardinalities, as obtained for whole-brain co-alteration. Colors are coherent with the sagittal views, which were also shown in Fig. 2. *C* = 5 is not shown since only the orange central node changed with respect to *c* = 4

The MDS showed a clear separation among the clusters at each of the cardinality levels considered, thus supporting the hierarchical clustering results (Fig. 4).

### Relationship with functional connectivity

The comparison of within-cluster and between-cluster Pearson correlation of the SVC maps confirmed that the former was always higher for each cluster up to the 5 clusters solution. Above that level of cardinality, this was no longer true for the fronto-parietal ensemble, although the only cluster to show a higher between-cluster correlation was spatially close to it and originated from its same fragmentation (Table 2 and Tables S2–S8).

**Table ﻿2:** Maximum Pearson correlation observed in each cluster for SVC maps

Cardinality	Cluster	*r* max	Within/between
2C	c1	**0.389**	Within
	c2	**0.262**	Within
3C	c1	**0.389**	Within
	c2	**0.956**	Within
	c3	**0.341**	Within
4C	c1	**0.308**	Within
	c2	**0.579**	Within
	c3	**0.956**	Within
	c4	**0.341**	Within
5C	c1	**0.308**	Within
	c2	**0.579**	Within
	c3	**0.956**	Within
	c4	**1.000**	Within
	c5	**0.402**	Within
6C	c1	**0.332**	Between, *c*2
	c2	**0.871**	Within
	c3	**0.825**	Within
	c4	**0.956**	Within
	c5	**1.000**	Within
	c6	**0.402**	within
7C	c1	**0.332**	Between, c2
	c2	**0.871**	Within
	c3	**0.825**	Within
	c4	**0.956**	Within
	c5	**1.000**	Within
	c6	**0.394**	Within
	c7	**0.534**	Within
8C	c1	**0.332**	Between, c2
	c2	**0.871**	Within
	c3	**0.921**	Within
	c4	**0.869**	Within
	c5	**0.956**	Within
	c6	**1.000**	Within
	c7	**0.394**	Within
	c8	**0.534**	Within

**Fig. 5:**
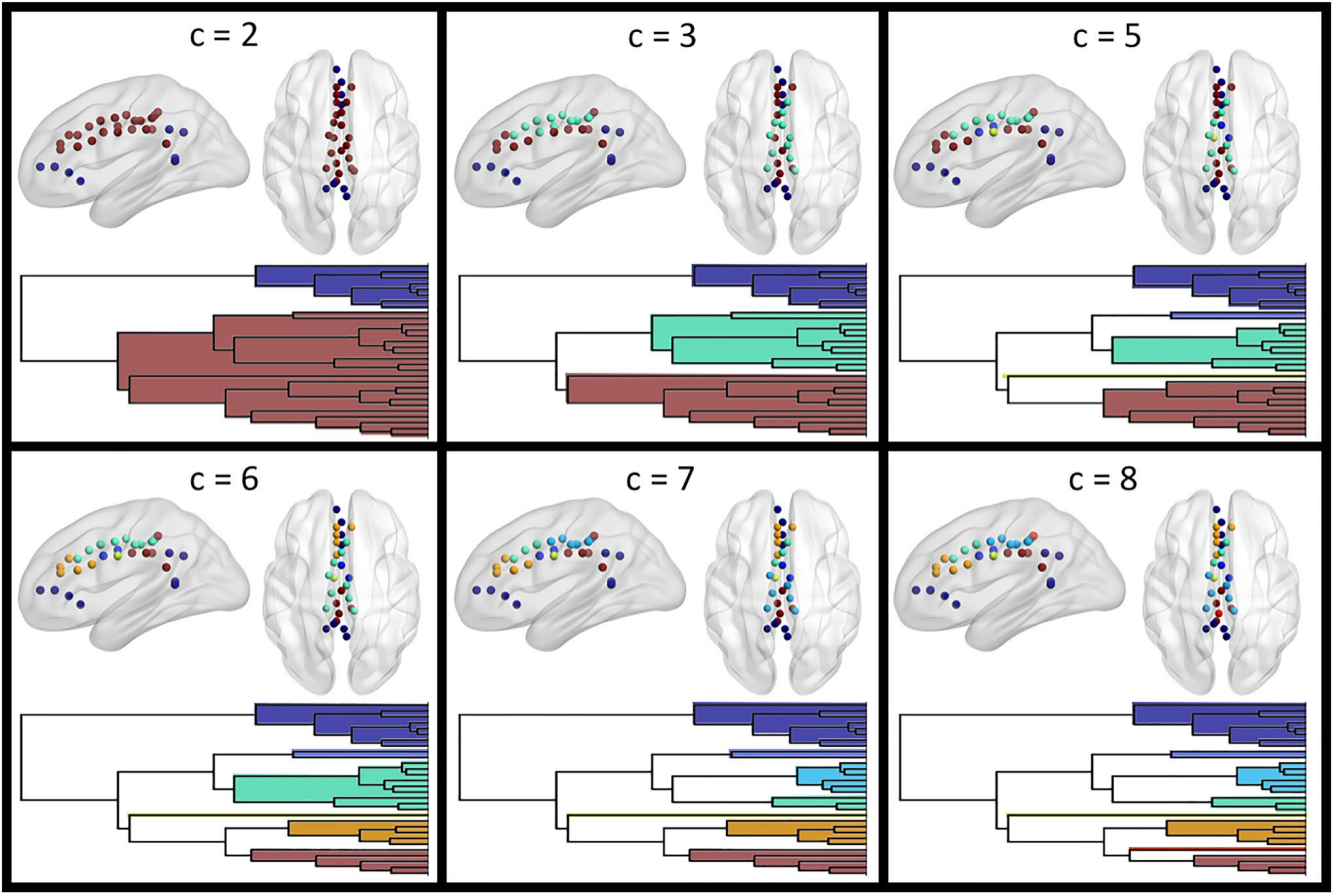
Results of the hierarchical clustering (Pearson correlation and WPGMA) of the SVC maps originating from each of the 30 root nodes. Axial views are in neurological convention (left is left). C = 4 is not shown since only the yellow middle node changed with respect to *c* = 3. Colors are coherent between the dendrogram and the visualization of the nodes

**Fig. 6:**
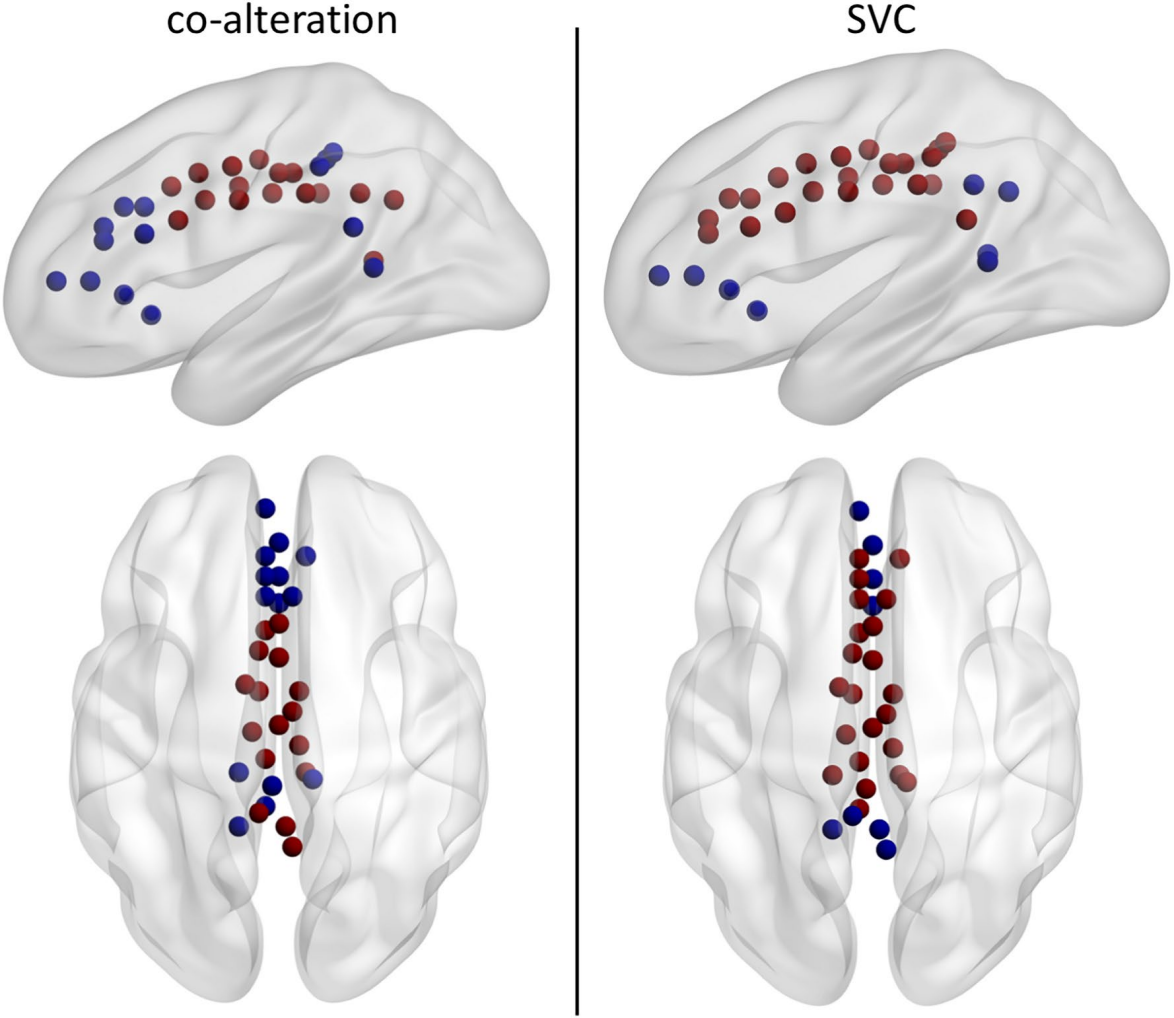
A comparison of the two clusters solution based on co-alteration (left) or SVC (right) at level *c* = 2. Axial views are in neurological convention (left is left)

The hierarchical clustering directly applied to the SVC maps (Fig. 5) confirmed the long-range ensemble, although now limited to the most rostral and caudal nodes (Fig. 6). Also in this case, the effect was still visible while increasing the number of clusters. Apart from this, the co-alteration and the SVC clustering were quite different, with the former showing a rostro-caudal gradient, while the latter a dorsal–ventral one.

### Functional network decomposition

**Fig. 7:**
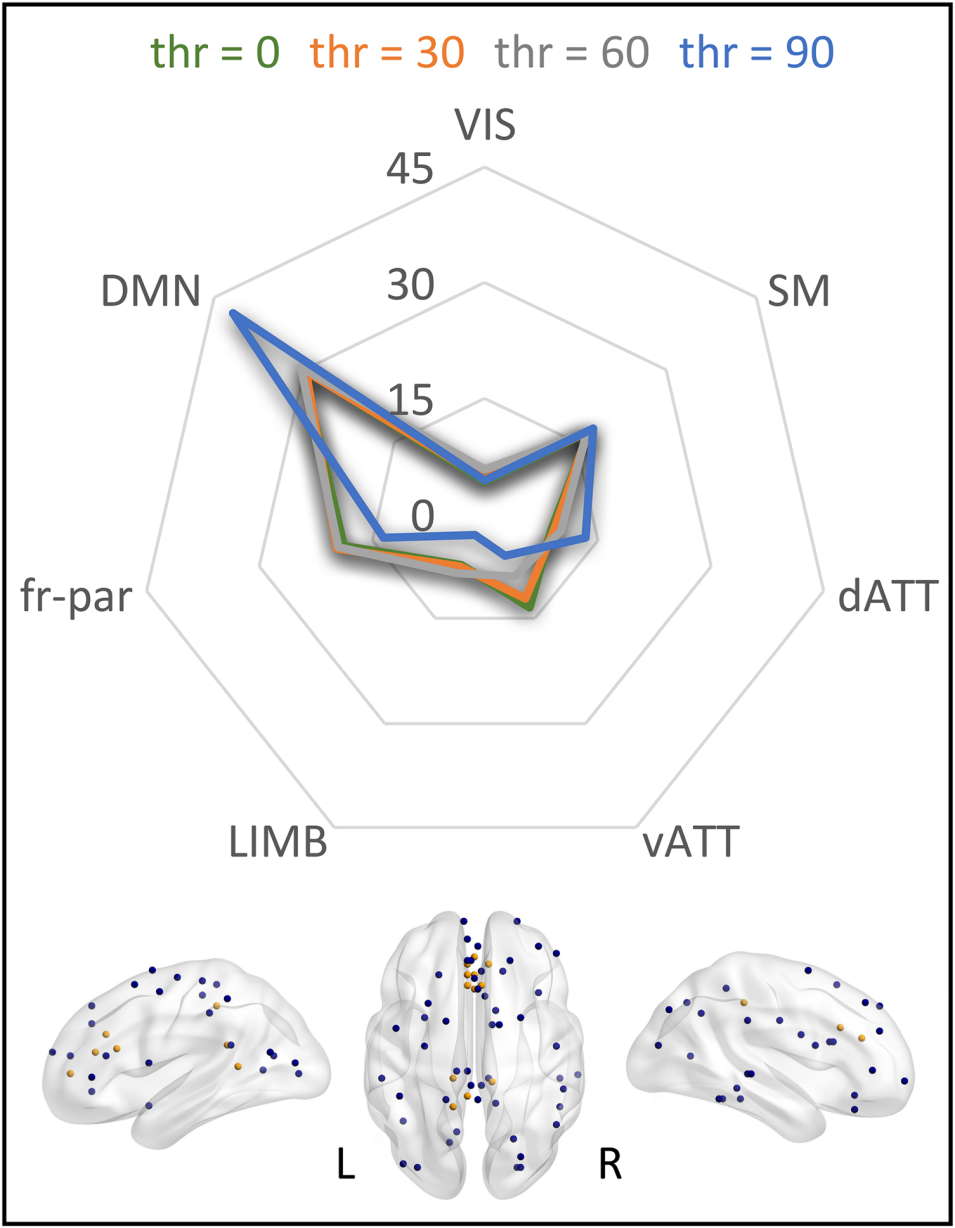
Results of the functional network decomposition for the fronto-parietal cluster. Top: Each line represents the thresholding on a different percentile of the Patel’s κ values distribution. Values express the percentage of nodes located in a network with respect to the total number of nodes. *VIS* visual, *SM* sensory-motor, *dATT* dorsal attentional, *vATT* ventral attentional, *LIMB* limbic, *fr-par* fronto-parietal, *DMN* default mode network. Bottom: visualization of the nodes with at least one edge above the threshold of the 90th percentile (i.e. data behind the blue line in the radar chart). Root nodes are colored in yellow. Axial view is in neurological convention (left is left)

The functional repartition of the sub-network composed of the first-neighbors of the fronto-parietal root nodes showed that the 30.3% of them was located in the DMN, making it the most represented among the resting state networks. This percentage reached the 41.8% when setting the threshold to the 90th percentile of the Patel’ *κ* values distribution (Fig. 7).

## Discussion

In the present meta-analytic work, we proposed a new methodology to identify sub-regions of the cingulate cortex based on their profile of structural co-alteration with the rest of the brain. The data-driven analysis of VBM data highlighted 30 nodes in the cingulate cortex (i.e. root-nodes), corresponding to peaks of high coherence among multiple experiments reporting pathological structural alteration. The root nodes were distributed across the whole cingulate cortex, with a prevalence in the left hemisphere. This leftward lateralization had been already described in the co-alteration of the hippocampus in Alzheimer’s disease (Manuello et al. 2018), as well as in the whole brain in autism spectrum disorder (Liloia et al. 2021b). The opposite was observed in the insular cortex (Nani et al. 2021). A whole brain, transdiagnostic meta-analysis suggested a differential involvement of the two hemispheres, the right one appearing to be more relevant for a MCN of decreases, while the left one for increases. Also, the interaction between the two forms of GM alteration in psychiatric disorders seemed to be more concentrated in the left hemisphere (Mancuso et al. 2020). Despite the major involvement of the left cingulate cortex, in the present work the results of the clustering procedure were mostly symmetrical between the hemispheres (Fig. 1). So, it seems that the lateralization of co-alteration patterns might depend on the area and possibly the pathology analyzed. Further investigation is needed to elucidate this phenomenon.

Contrary to other clustering techniques, the hierarchical one does not recommend a preferable cardinality. It is therefore equally reasonable to analyze each level of the obtained dendrogram, with the advantage of focusing on multiple levels of resolution. In this specific case, this was further supported by the MDS results that showed a good separation of the clusters on multiple cardinalities. The highest branch (i.e. *c* = 2) yielded unpredicted results, showing a fronto-parietal cluster. In fact, a bipartite solution could have been expected to match Brodmann’s model along the antero-posterior axis, or at least to follow a criterion of spatial contiguity, as mostly was for the remaining clusters. However, the observed pattern can be associated with the DMN. Indeed, sub-regions of this network are known to be structurally altered in several diseases (Liloia et al. 2021b; Manuello et al. 2018), and it is therefore not surprising to find its involvement when adopting a cross-disorders approach. Coherently with our results, physically distant anterior and posterior portions of the cingulate cortex had been already found to be grouped in a same cluster in the work of Torta et al. (2013), although based on task-fMRI data. It is interesting to note that this fronto-parietal cluster is the first to be formed during the linkage iterative process, when the most similar nodes are identified and combined. Therefore, this is not the case of two independent clusters being merged at a later stage. Similarly, the observed development of the dendrogram seems to exclude the case of a cluster collecting outliers not fitting elsewhere. Notably, the distribution we found proved to be unaffected by technical parameters, such as the linkage method.

Since local and long-range co-alteration were shown to have a different contribution to the development of MCNs (Cauda et al. 2020), it was relevant to detail the role of co-alteration within the cingulate cortex in shaping the obtained dendrogram. Although even the smallest Patel’s *κ* value is statistically significant, we decided to describe edges exceeding 0.5 to focus on the strongest evidence. Interestingly, different patterns were observed depending on the sub-section of this region. A tight net of short edges characterized the nodes located in the middle cingulate cortex. This is coherent with the presence of the two wide clusters already visible at *c* = 8 and merging into one at *c* = 6. In light of this, it is possible to deduce that the middle portion of the cingulate cortex behaves as a rather homogeneous entity in brain pathologies. This trend did not propagate to the posterior section, where the small cluster of two nodes remained isolated from the rest of the network. Long-range projections characterized the fronto-parietal ensemble instead, with the node in the retrosplenial cortex joining the frontal and parietal cliques. The literature on alteration of the retrosplenial cortex in brain disorders is rather limited. A form of retrosplenial amnesia is known (Aggleton 2010; Valenstein et al. 1987), and not surprisingly some authors described its involvement in Alzheimer’s disease (Maass et al. 2017), although in conjunction with anterior thalamic nuclei rather than other sections of the cingulate cortex (Aggleton et al. 2016). The interpretation of the local co-alteration of the cingulate cortex cannot exclude the interplay with the cingulate bundle (Bubb et al. 2018). As in the case of cingulate cortex, there is no consensus of the subdivision of the fiber bundle, with the finer grained solutions proposing at least 5 distinct tracts (Heilbronner and Haber 2014; Vogt 2009). This evidence is coherent with our identification of different clusters supported by local co-alteration. Notably, even the schemata reporting a lower number of subdivisions in the cingulate bundle distinguished a retrosplenial tract (Budisavljevic et al. 2016; Jones et al. 2013). If, on the one hand, this supports the differential behavior observed for the root node in the retrosplenial cortex, on the other hand this does not explain the long-range edges linking the frontal and parietal root nodes. This could be propped up at least in part by the thalamocingulate tract (Weininger et al. 2019) although in spite of its first description almost one century ago (Clark and Boggon 1933; Waller 1934) there is still controversy about its termination in humans (see Weininger et al. (2019) for a review of anatomical findings).

According to our results, the fronto-parietal cluster is not exclusively supported by local co-alteration. In fact, when the edges between root-nodes were removed that ensemble was still visible, suggesting its involvement in a common network of brain regions being co-altered with most rostral and caudal portion of the cingulate-cortex. Previous evidence from pathoconnectomics showed that both structural and functional connectivity have a role in shaping the MCNs (Cauda et al. 2020, 2018; Liloia et al. 2021b), in line with computational studies supporting models of network spread of pathology for degeneration disorders (Raj and Powell 2018; Zhou et al. 2012). In this sense, the functional repartition confirmed the possible involvement of canonical resting state networks, in particular of the DMN. Notably, increasing the Patel’s *κ* threshold, which means focusing on brain regions with a higher probability of co-alteration, specifically selected nodes in the DMN. Further defining this network involving the fronto-parietal cluster, in particular determining the directionality of the propagation of the alteration within it, could be a meaningful focus of future research in the field of pathoconnectomics.

Looking at the solution with 4 clusters, it showed good coherence with the four-region neurobiological model proposed by Vogt et al. (2004) and further validated by Palomero-Gallagher et al. (2009). The identification of the dorsal posterior cluster consisting of two nodes (already visible in the 3 clusters solution) could correspond with the neurochemical distinction between Brodmann area 31 and the other areas composing the posterior cingulate cortex in Palomero-Gallagher et al. (2009). The distinction of those 4 clusters was also supported by the analysis of local co-alteration within the nodes in the cingulate cortex.

Further increasing the number of clusters, our solution did not seem to approximate the 6 sub-regions functional model proposed by Jin et al. (2018). In particular, the co-alteration based parceling did not highlight dorsal–ventral distinctions. Higher similarity can be found instead between our 8 clusters solution and the anatomical subdivision into 10 sub-regions by Jin et al. (2018), especially for their S1–S2–S3 organization of the anterior cingulate cortex. The trend of fragmentation we observed in the anterior portion of the cingulate cortex could reflect the electrophysiological results by Caruana et al. (2018). Across the various cardinalities, the MDS suggest a different behavior in structural co-alteration between the central portion of the cingulate cortex and the fronto-temporal regions.

The complementary clustering of the SVC maps of the root-nodes, together with the comparison of within-cluster and between-clusters correlation, further consolidated the hypothesized role of functional connectivity. In fact, the fronto-parietal cluster observed in the co-alteration model was evident from the first branching of the functional dendrogram, and was preserved intact at finer resolution. Interestingly, this ensemble now also included the two posterior nodes, which were differentiated instead in the co-alteration based dendrogram, and coherently segregated by local co-alteration. On the contrary, 3 nodes in the posterior section of the cingulate cortex are no longer part of the fronto-parietal cluster, showing instead considerable heterogeneity across the dendrogram. Also in the anterior part of the cingulate cortex, an ensemble of 5 nodes changed allocation. As pointed out by Cauda et al. (2018) the weighted contribution of different connectivity modalities to the development of the MCNs is likely to change depending on the pathology investigated. Recently, Nani et al. (2021) highlighted that in the insula the pattern of co-alteration does not tightly follow healthy functional connectivity. Our results suggest that this kind of differences could be appreciated even at the level of sub-structures, as in the case of the posterior nodes being assigned to different cluster depending on the modality considered. Therefore, the co-alteration based approach here proposed could allow to open a yet unexplored level of analysis which allows to describe pathological patterns directly from modality-specific data, being at the same time coherent with functional and structural connectivity of the healthy brain, and potentially resolving the possible conflict between them.

### Limitations and future directions

As in any coordinate-based meta-analysis, it is not possible to exclude that, despite literature screening, relevant publications were missed. However, the use of the BrainMap environment ensured standardized search criteria and facilitated reproducibility (Vanasse et al. 2018). A further limitation deriving from the adoption of a cross-disorder point of view is the inability to distinguish how different brain disorders impact on the cingulate cortex and its sub-regions. On the other hand, this approach allowed to take into account different pathological processes affecting this structure. At the same time, the bigger the analyzed sample of studies is the more robust the statistical outcome. Indeed, the present work was intended to provide useful baseline for further examinations with more specific aims. For example, the observed sub-regions of the cingulate cortex could be used to analyze single pathologies, or as seeds for connectivity studies. Finally, the Patel’s κ does not allow to assess whether alterations first originate in the cingulate cortex and then propagate to other regions or vice versa. Future consideration of directionality, both spatial and temporal, will likely improve our comprehension of the structural co-alteration phenomenon.

## Conclusion

We described an innovative approach to investigate the organization of the brain based on pathological co-alteration, and applied it to parcel the cingulate cortex. Through this methodology, we identified a fronto-parietal pattern that suggests the involvement of the DMN. Within it, the analysis of local co-alteration highlighted an intriguing interplay between the retrosplenial cortex and the anterior and posterior cingulate cortex, so far little described in the literature. The use of hierarchical clustering allowed a multi-level description of our findings, overall confirming the existence of an interplay between anatomical and functional properties and co-alteration. In conclusion, the proposed methodology could open a new window onto the pathological brain, allowing to elucidate mechanisms behind it together with its structural properties.

## Supplementary Information

Below is the link to the electronic supplementary material.

Supplementary file1 (PDF 2514 KB)

## Data Availability

Meta-analytic data used in the present work can be freely retrieved from the BrainMap database. The voxel-wise parceling can be freely downloaded from figshare https://doi.org/10.6084/m9.figshare.16708816.v1. The AAL atlas can be freely downloaded from https://www.gin.cnrs.fr/en/tools/aal/.
