# Supplementary material for: A co-alteration parceling of the cingulate cortex
Source: Brain Struct Funct. 2022 Mar 3;227(5):1803–16. doi: 10.1007/s00429-022-02473-2 (PMC9098570; doi:10.1007/s00429-022-02473-2)
Supplement: Supplementary file 1 — Supplementary file1 (PDF 2514 KB) [file 429_2022_2473_MOESM1_ESM.pdf]

A co-alteration parceling of the cingulate cortex

Jordi Manuello[1,2], Lorenzo Mancuso[2], Donato Liloia[1,2], Franco Cauda[1,2,3], Sergio Duca[1,2], Tommaso Costa[1,2]

[1] GCS fMRI, Koelliker Hospital and University of Turin, Turin, Italy

[2] FOCUS Lab, Department of Psychology, University of Turin, Turin, Italy

[3] Neuroscience Institute of Turin, Turin, Italy

Supplementary Materials

Supplementary methods…p. 2

Supplementary figures…p. 4

Supplementary tables…p. 8

Supplementary methods

1 Data search
The PRISMA Statement guidelines (Liberati et al., 2009; Moher et al., 2009) were adopted to ensure a transparent report of data selection, screening and extraction steps.

1.1 Identification phase
A reproducible search was employed to extract experimental meta-data of interest, published until March 2021, across the voxel-based morphometry (VBM) database of BrainMap (Fox et al., 2005; Fox and Lancaster, 2002; Laird et al., 2005; Vanasse et al., 2018). BrainMap comprises an open access repository of over 4,400 neuroimaging articles (functional and structural MRI published findings), from which data on regional effects can be retrieved. In order to assess the impact of brain disorders on the cingulate cortex we constructed a standardized search algorithm using the software application *Sleuth 3.0.4.* (http://www.brainmap.org/sleuth/), capable of retrieving all the VBM experiments that matched the following query:

[Experiments Contrast is Gray Matter] AND [Experiment Context is Disease] AND [Observed Changes is Controls>Patients] AND [Locations MNI image is cingulate_cortex_mask]

1.2 Screening and eligibility phases
Our data-driven selection retrieved 193 experiments, 4941 clinical subjects and 2985 coordinates of alteration (see also Figures S1 and Table S1). Of note, all the selected experiments were reviewed in order to ensure:

1) they were published in a peer-reviewed English language article;
2) that the experiments described gray matter morphometric changes (i.e., volume and/or concentration) visible with VBM method;
3) both the presence of the healthy control group and the clinical sample;
4) that the results were reported by using a stereotactic space (i.e., Talairach/Tournoux or Montreal Neurological Institute).

The coordinate-based meta-analysis was carried out in the Talairach (TAL) stereotactic space. Coordinates (x-y-z) reported in MNI space were converted into TAL using the icbm2tal algorithm (Lancaster et al., 2007).

1.4 Inclusion
For the overview of the selection strategy see Figure S1 (PRISMA flow chart). For the distribution of the selected experiments see also Table S1.

References
Fox, P.T., Laird, A.R., Fox, S.P., Fox, P.M., Uecker, A.M., Crank, M., Koenig, S.F., Lancaster, J.L., 2005. BrainMap taxonomy of experimental design: description and evaluation. Hum Brain Mapp 25, 185-198.
Fox, P.T., Lancaster, J.L., 2002. Opinion: Mapping context and content: the BrainMap model. Nat Rev Neurosci 3, 319-321.

Laird, A.R., Lancaster, J.L., Fox, P.T., 2005. BrainMap: the social evolution of a human brain mapping database. Neuroinformatics 3, 65-78.
Lancaster, J.L., Tordesillas-Gutierrez, D., Martinez, M., Salinas, F., Evans, A., Zilles, K., Mazziotta, J.C., Fox, P.T., 2007. Bias between MNI and Talairach coordinates analyzed using the ICBM-152 brain template. Hum Brain Mapp 28, 1194-1205.
Liberati, A., Altman, D.G., Tetzlaff, J., Mulrow, C., Gotzsche, P.C., Ioannidis, J.P., Clarke, M., Devereaux, P.J., Kleijnen, J., Moher, D., 2009. The PRISMA statement for reporting systematic reviews and meta-analyses of studies that evaluate health care interventions: explanation and elaboration. J Clin Epidemiol 62, e1-34.
Moher, D., Liberati, A., Tetzlaff, J., Altman, D.G., 2009. Preferred reporting items for systematic reviews and meta-analyses: the PRISMA statement. J Clin Epidemiol 62, 1006-1012.
Rosenthal, R., 1979. The file drawer problem and tolerance for null results. Psychological Bulletin 86, 638-641.
Vanasse, T.J., Fox, P.M., Barron, D.S., Robertson, M., Eickhoff, S.B., Lancaster, J.L., Fox, P.T., 2018. BrainMap VBM: An environment for structural meta-analysis. Hum Brain Mapp 39, 3308-3325.

Supplementary Figures

Figure S1. PRISMA flow chart for the meta-data selection strategy.

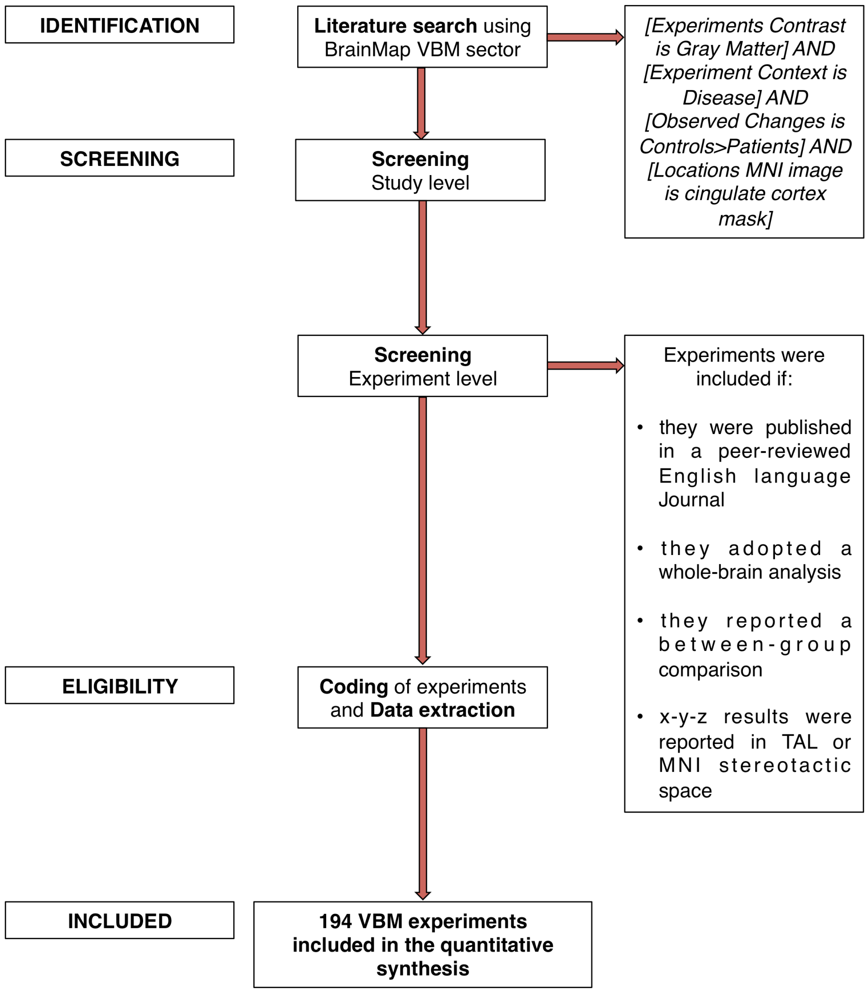

Figure S2. Sagittal slices of the ROI used to define the cingulate cortex in the present work, based on the AAL atlas (version 3.1). This includes 6308 voxels with 2mm resolution.

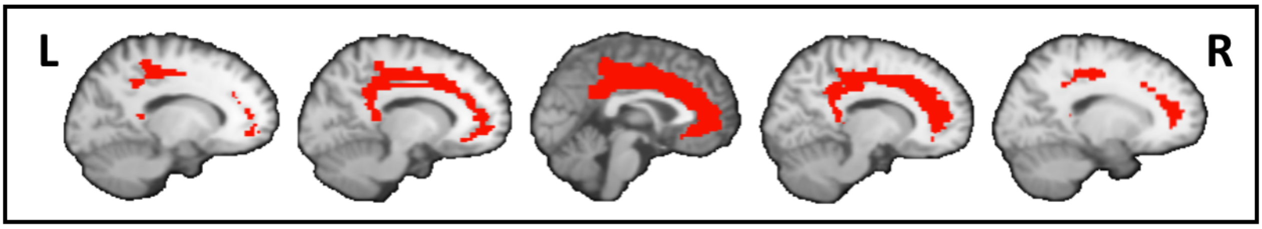

Figure S3. The dendrogram obtained through hierarchical clustering (using Pearson correlation and WPGMA) cut at c=20. The blue cluster consists of the fronto-parietal root nodes. Each of the 16 uncolored clusters includes one root node only.

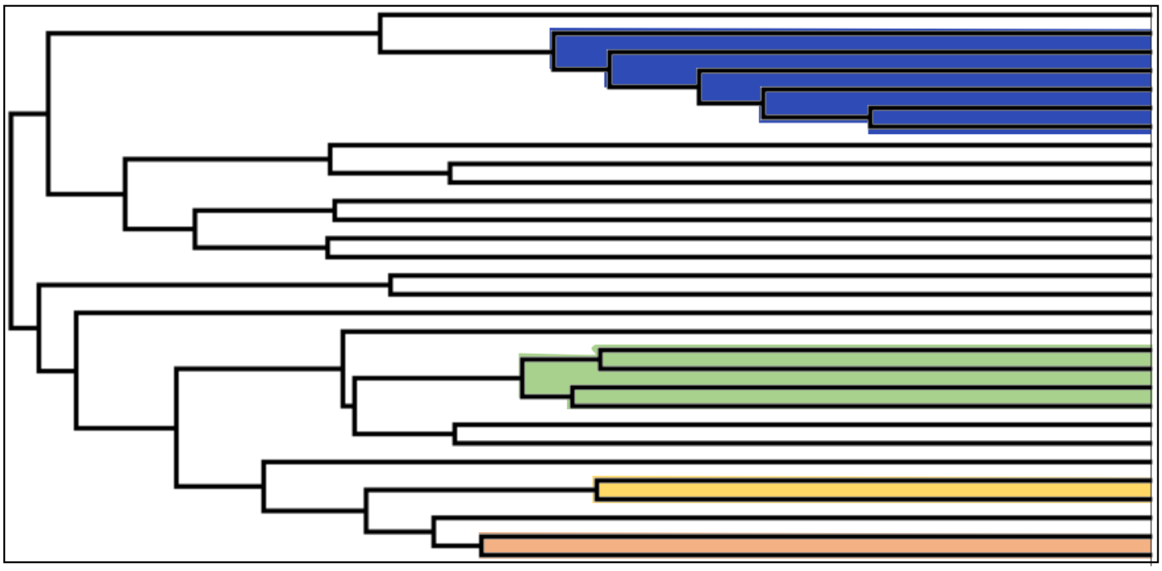

Figure S4. Visualization of the 5 first root nodes (in blue) to be clustered together in the dendrogram (using Pearson correlation and WPGMA). At this stage, the red nodes are not yet assigned to a cluster.

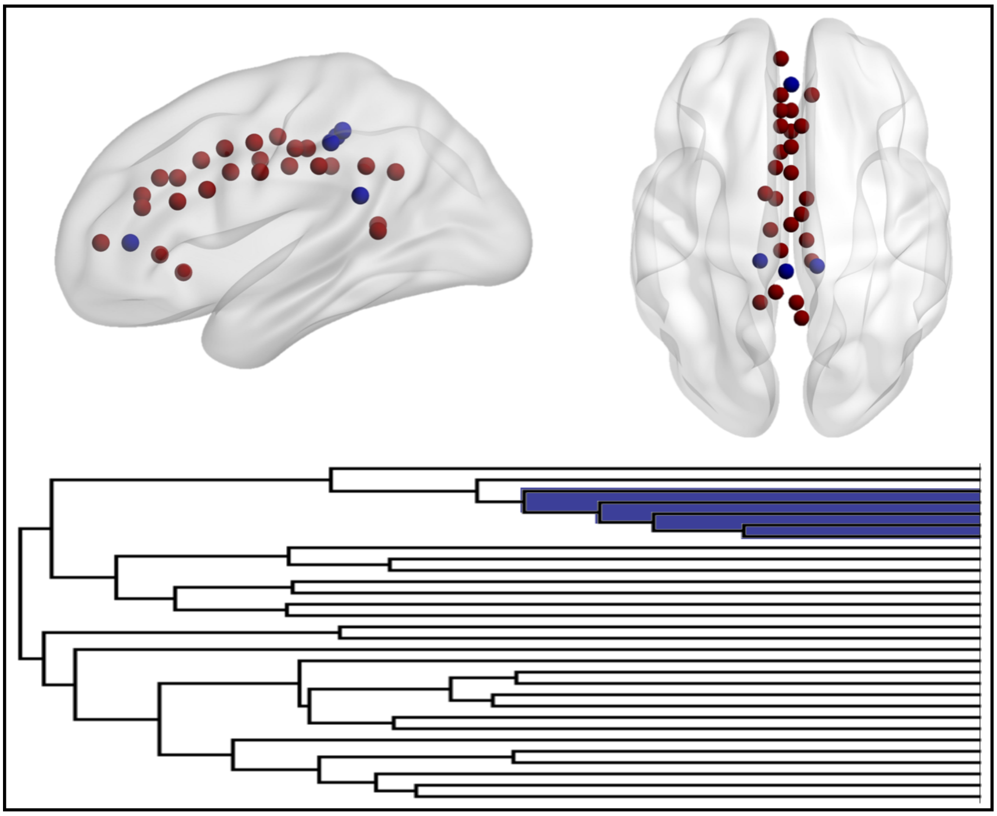

Figure S5. Comparison of the c=2 solution obtained with different linkage methods: WPGMA (left), average (middle), complete (right). Pearson correlation was used as distance metric in each condition.

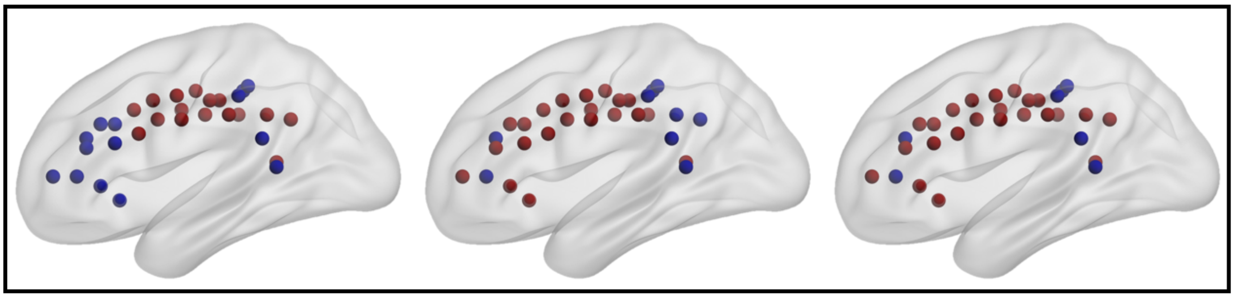

Figure S6. Sagittal view of the voxel-wise parceling at different cardinalities.

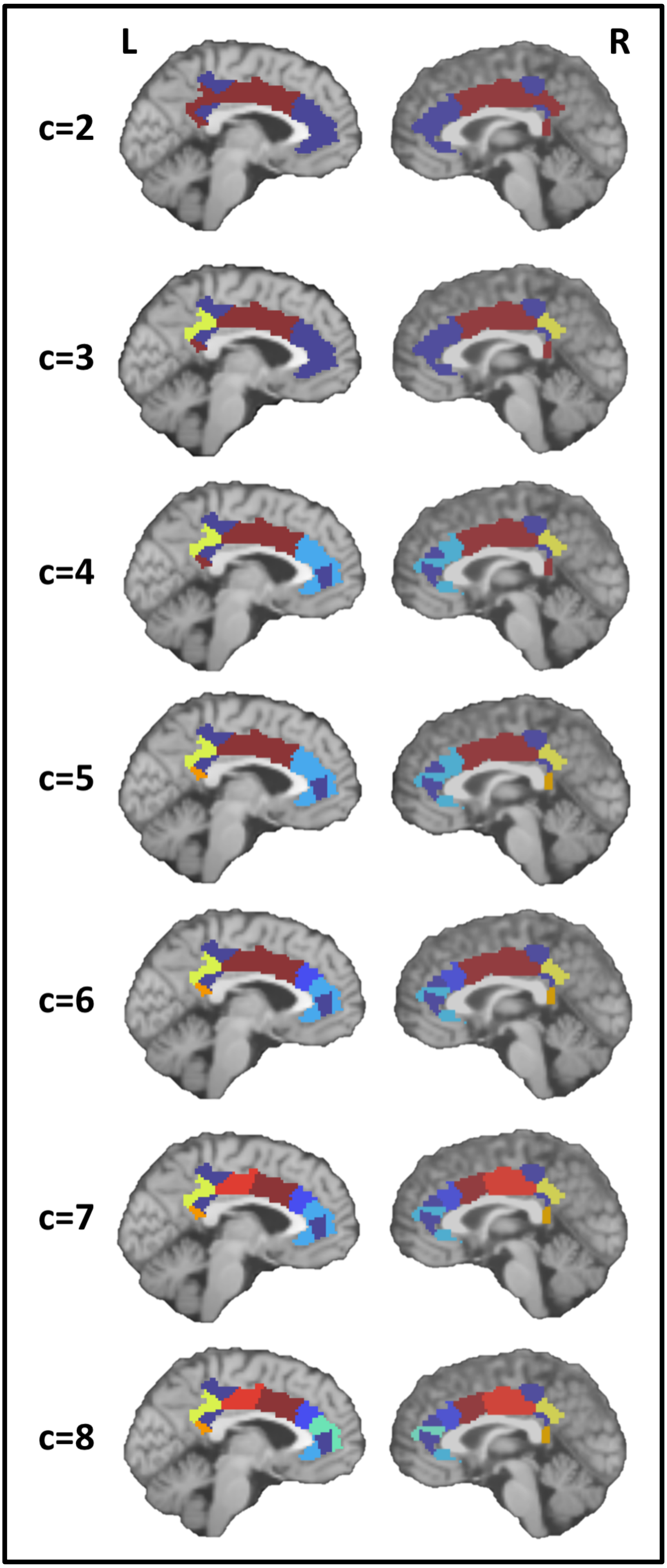

Supplementary Table

Table S1. VBM experiments included in the coordinate-based meta-analysis. The records shown in the table are the result of the data-driven selection process as shown in the PRISMA flow chart (Figure S1) and in the supplementary methods section. The starting point for the selection can be traced in the algorithms and in the additional considerations previously proposed. For the articles including independent groups with the same main diagnosis, the specific classification was provided.

First Author	***Year***	***Subj (N)***	***Foci (N)***	***Clinical Condition***
Abe O	2010	21	9	Major depressive disorder
Agosta F	2010	10	16	Progressive supranuclear palsy
Agosta F	2011	15	3	Alzheimer's disease
Ahmed F	2012	21	3	Post-traumatic stress disorder
Arnone D	2009	25	4	Major depressive disorder
As-Sanie S	2012	17	6	Chronic pain
Asami T	2009	9	14	Panic disorder
Ash S	2011	11	43	Lewy body spectrum disorder
Ash S	2009	9	7	Frontotemporal lobar degeneration
Audoin B	2010	37	26	Multiple sclerosis
Aydin K	2009	17	11	Subacute sclerosing panencephalitis
Barad M J	2013	15	6	Complex regional pain syndrome
Barbeau E	2008	16	13	Mild cognitive impairment
Baron J C	2001	16	50	Alzheimer's disease
Baxter L C	2006	15	6	Alzheimer's disease
Bernasconi N	2004	45	26	Temporal lobe epilepsy (left side)
Bernasconi N	2004	40	13	Temporal lobe epilepsy (right side)
Beyer M K	2007	16	18	Parkinson disease
Bitter T	2010	17	35	Anosmia
Bitter T	2010	24	15	Hyposmia
Boccardi M	2005	9	19	Frontotemporal dementia
Bonath B	2018	18	12	Attention deficit hyperactivity disorder
Borgwardt S J	2010	9	10	Schizophrenia
Boxer A L	2006	14	11	Corticobasal degeneration syndrome
Bozzali M	2006	20	14	Mild cognitive impairment
Brazdil M	2009	20	8	Temporal lobe epilepsy
Brenneis C	2004	12	12	Progressive supranuclear palsy
Brooks S J	2011	6	5	Anorexia nervosa
Canu E	2010	24	22	Alzheimer's disease (late onset)
Canu E	2010	18	15	Alzheimer's disease (early onset)
Chang J L	2005	10	14	Amyotrophic lateral sclerosis
Chang S E	2008	7	14	Stuttering
Chanraud S	2007	28	10	Alcohol dependent
Chanraud S	2009	24	14	Alcohol dependent
Chen S	2006	12	4	Post-traumatic stress disorder
Chen S	2009	12	3	Post-traumatic stress disorder
Chen Y	2012	10	1	Post-traumatic stress disorder
Chua S E	2007	26	10	Schizophrenia

Corbo V	2005	14	5	Post-traumatic stress disorder
Cordato N J	2005	21	9	Progressive supranuclear palsy
Craig M C	2007	14	5	Autism spectrum disorder
Critchley H D	2003	15	10	Pure autonomic failure
De Oliveira-Souza R	2008	15	22	Psychopathy
Di Paola M	2007	18	18	Alzheimer's disease
Doris A	2004	11	33	Bipolar disorder
Douaud G	2007	25	23	Schizophrenia
Draganski B	2006	28	6	Limb amputation
Draganski B	2003	10	3	Idiopatic cervical dystonia
Eckart C	2011	13	5	Post-traumatic stress disorder
Ecker C	2010	22	14	Autism spectrum disorder
Eshaghi A	2014	19	6	Multiple sclerosis
Fahim C	2012	8	1	Oppositional defiant disorder
Feldmann A	2008	1	7	Posterior cortical atrophy
Friedrich H C	2012	12	8	Anorexia nervosa
Frisoni G B	2002	26	34	Alzheimer's disease
Frodl T	2008	30	65	Major depressive disorder
Gale S D	2005	9	16	Traumatic brain injury
Gaudio S	2011	16	3	Anorexia nervosa
Giuliani N R	2005	34	14	Schizophrenia
Gobbi C	2014	54	56	Multiple sclerosis (non-depressed)
Gobbi C	2014	69	56	Multiple sclerosis (depressed)
Gobbi C	2014	59	56	Multiple sclerosis (non-fatigued)
Gobbi C	2014	64	56	Multiple sclerosis (fatigued)
Greimel E	2013	47	4	Autism spectrum disorder
Grieve S M	2013	34	41	Major depressive disorder
Guggenmos M	2017	97	22	Alcohol dependent
Ha T H	2010	23	18	Bipolar disorder
Ha T H	2004	35	13	Schizophrenia
He N	2015	35	4	Attention deficit hyperactivity disorder
Henley S M	2009	20	27	Huntington disease
Herold R	2009	18	38	Schizophrenia
Hirao K	2008	20	6	Schizophrenia
Honea R A	2009	56	13	Alzheimer's disease
Horn H	2009	13	12	Schizophrenia
Huang C W	2018	30	5	Alzheimer's disease
Hulshoff Pol H E	2001	158	25	Schizophrenia
Jang D P	2007	20	12	Alcohol dependent
Janssen J	2008	20	1	Bipolar disorder
Joo E Y	2010	31	27	Obstructive sleep apnea syndrome
Joos A	2010	12	7	Anorexia nervosa
Kanda T	2008	13	9	Frontotemporal dementia
Kanda T	2008	20	7	Alzheimer's disease
Kasai K	2008	18	7	Post-traumatic stress disorder
Kasparek T	2010	49	17	Schizophrenia
Kassubek J	2007	18	10	Kennedy disease

Kawachi T	2006	30	14	Alzheimer's disease
Kawada R	2009	26	13	Schizophrenia
Kawasaki Y	2004	25	18	Schizophrenia
Keller S S	2002	40	4	Temporal lobe epilepsy (left side)
Keller S S	2002	36	4	Temporal lobe epilepsy (right side)
Keller S S	2004	40	6	Hippocampal atrophy (left side)
Keller S S	2004	36	6	Hippocampal atrophy (right side)
Kim J H	2008	20	20	Migraine
Kim S J	2009	17	29	Narcolepsy
Koening P	2008	6	8	Alzheimer's disease
Koutsouleris N	2008	59	26	Schizophrenia (negative symptoms)
Koutsouleris N	2008	61	20	Schizophrenia (positive symptoms)
Koutsouleris N	2008	55	16	Schizophrenia (disorganized symptoms)
Kroes M C W	2010	24	3	Post-traumatic stress disorder/Major depressive disorder
Kubicki M	2002	16	9	Schizophrenia
Kuchinad A	2007	10	5	Fibromyalgia
Lagarde J	2013	16	8	Frontotemporal dementia
Lai C H	2010	15	12	Major depressive disorder
Lai M	2013	30	1	Autism spectrum disorder
Leung K K	2009	17	11	Major depressive disorder
Li L	2006	12	4	Post-traumatic stress disorder
Lin C H	2013	10	15	Parkinson disease
Lin C H	2013	10	18	Essential tremor
Lochhead R A	2004	4	3	Bipolar disorder
Lu C	2010	12	9	Stuttering
Lyoo I K	2004	39	4	Bipolar disorder
Mak A K	2009	17	7	Major depressive disorder
Maneru C	2003	13	9	Hippocampal hypoxic-ischemic encephalopathy
Matsuda H	2002	15	20	Alzheimer's disease (illness duration 1 year)
Matsuda H	2002	15	23	Alzheimer's disease (illness duration 2 years)
Matsumoto R	2010	16	4	Obsessive-compulsive disorder
McAlonan G M	2005	17	13	Autism spectrum disorder
McMillan A B	2004	12	14	Temporal lobe epilepsy
Meda S A	2008	133	51	Schizophrenia (JHU sample)
Meda S A	2008	34	37	Schizophrenia (MPRC sample)
Meisenzahl E M	2008	93	48	Schizophrenia (first-episode)
Meisenzahl E M	2008	72	67	Schizophrenia (recurrently ill)
Mengotti P	2011	20	2	Autism spectrum disorder
Mesaros S	2008	21	64	Multiple sclerosis
Minnerop M	2007	32	18	Multiple system atrophy (cerebellar)
Minnerop M	2007	16	22	Multiple system atrophy (parkinsonian)
Narita K	2011	14	10	Bipolar disorder
O'Muircheartaigh J	2011	28	2	Juvenile myoclonic epilepsy
Ohnishi T	2006	19	19	Schizophrenia
Pennanen C	2005	32	10	Mild cognitive impairment
Pereira J B	2009	20	30	Parkinson disease
Pereira J M	2009	9	3	Frontotemporal dementia (group 1)

Pereira J M	2009	6	4	Frontotemporal dementia (group 2)
Pereira J M	2009	4	2	Frontotemporal dementia (group 3)
Prakash R S	2010	15	12	Multiple sclerosis
Prinster A	2006	34	8	Multiple sclerosis
Prinster A	2010	35	20	Multiple sclerosis
Pujol J	2004	72	3	Obsessive-compulsive disorder
Quarantelli M	2006	30	9	Facioscapulohumeral dystrophy
Rabinovici G D	2007	18	38	Frontotemporal dementia
Rami L	2009	27	6	Alzheimer's disease
Redlich R	2014	58	8	Major depressive disorder
Redlich R	2014	58	8	Bipolar disorder
Riederer F	2008	12	10	Temporal lobe epilepsy (left side)
Riederer F	2012	29	8	Medication-overuse headache
Ries M L	2009	15	9	Major depressive disorder
Rocca M A	2006	15	21	Migraine
Rocca M A	2014	31	12	Multiple sclerosis
Rodriguez-Raecke R	2009	32	16	Osteoarthritis
Roman-Urrestarazu A	2016	34	2	Attention deficit hyperactivity disorder
Rossi R	2012	14	38	Bipolar disorder
Rowe J B	2010	82	3	Parkinson disease
Ruscheweyh R	2011	31	26	Chronic pain
Salgado-Pineda P	2003	13	15	Schizophrenia
Salgado-Pineda P	2004	14	38	Schizophrenia
Salvadore G	2011	27	3	Major depressive disorder
Schmidt-Wilcke T	2010	11	9	Persistent idiopathic facial pain
Schmidt-Wilcke T	2008	31	4	Migraine
Schmidt-Wilcke T	2005	20	16	Chronic tension type headache
Seeley W W	2008	15	29	Frontotemporal dementia (group 1)
Seeley W W	2008	15	33	Frontotemporal dementia (group 2)
Seeley W W	2008	15	44	Frontotemporal dementia (group 3)
Serra-Blasco M	2013	22	12	Major depressive disorder
Sowell E R	2001	7	17	Fetal alcohol syndrome/prenatal alcohol exposure
Spano B	2010	10	12	Multiple sclerosis
Sukuzi M	2002	22	2	Schizophrenia (male)
Sukuzi M	2002	20	2	Schizophrenia (female)
Summerfield C	2005	13	10	Parkinson disease
Tanabe J	2009	19	1	Substance dependence
Tian L	2011	30	50	Schizophrenia
Tiihonen J	2008	25	31	Antisociality personality disorder
Tost H	2010	15	10	Bipolar disorder
Valet M	2009	14	13	Chronic pain
Valfre W	2008	27	10	Migraine
van Tol M J	2010	65	2	Major depressive disorder
Vannorsdall T D	2010	14	6	Traumatic brain injury
Vartiainen N	2009	8	7	Herpex simplex virus
Voets N L	2008	25	9	Schizophrenia
Weber Y G	2010	14	17	Myotonic dystrophy (type 1)

Weber Y G	2010	9	15	Myotonic dystrophy (type 2)
Whitford T J	2006	41	14	Schizophrenia
Whitwell J L	2005	7	6	Frontotemporal dementia
Whitwell J L	2013	16	4	Progressive supranuclear palsy
Wolf R C	2008	14	9	Schizophrenia
Wood P B	2009	20	4	Fibromyalgia
Xu L	2009	120	55	Schizophrenia
Yasuda C L	2010	34	23	Epilepsy (seizure-free)
Yasuda C L	2010	10	30	Epilepsy (no improvement)
Yatham L N	2007	15	3	First-episode mania
Yin C	2014	11	5	Mild cognitive impairment
Yoo S Y	2008	47	5	Obsessive-compulsive disorder
Zhang T	2009	15	4	Major depressive disorder
Zhao Y	2019	36	6	Attention deficit hyperactivity disorder

Table S2. Average Pearson correlation among SVC maps associated with the 30 root nodes, referred to the solution with 2 clusters. Within-cluster values are reported in the diagonal of the table, while off-diagonal values refer to between-cluster correlation. The highest value in each row (i.e. for each cluster) is highlighted in bold. Cluster numbers go from the top to the bottom of the dendrogram showed in Figure 2.

	**c1**	**c2**
**c1**	**0.389**	0.219
**c2**	0.219	**0.262**

Table S3. Average Pearson correlation among SVC maps associated with the 30 root nodes, referred to the solution with 3 clusters. Within-cluster values are reported in the diagonal of the table, while off-diagonal values refer to between-cluster correlation. The highest value in each row (i.e. for each cluster) is highlighted in bold. Cluster numbers go from the top to the bottom of the dendrogram showed in Figure 2.

	**c1**	**c2**	**c3**
**c1**	**0.389**	0.321	0.210
**c2**	0.321	**0.956**	0.198
**c3**	0.210	0.198	**0.341**

Table S4. Average Pearson correlation among SVC maps associated with the 30 root nodes, referred to the solution with 4 clusters. Within-cluster values are reported in the diagonal of the table, while off-diagonal values refer to between-cluster correlation. The highest value in each row (i.e. for each cluster) is highlighted in bold. Cluster numbers go from the top to the bottom of the dendrogram showed in Figure 2.

	**c1**	**c2**	**c3**	**c4**
**c1**	**0.308**	0.306	0.289	0.249
**c2**	0.306	**0.579**	0.279	0.239
**c3**	0.289	0.279	**0.956**	0.226
**c4**	0.249	0.239	0.226	**0.341**

Table S5. Average Pearson correlation among SVC maps associated with the 30 root nodes, referred to the solution with 5 clusters. Within-cluster values are reported in the diagonal of the table, while off-diagonal values refer to between-cluster correlation. The highest value in each row (i.e. for each cluster) is highlighted in bold. Cluster numbers go from the top to the bottom of the dendrogram showed in Figure 2.

	**c1**	**c2**	**c3**	**c4**	**c5**
**c1**	**0.308**	0.306	0.289	0.283	0.248
**c2**	0.306	**0.579**	0.279	0.273	0.237
**c3**	0.289	0.279	**0.956**	0.260	0.223
**c4**	0.283	0.273	0.260	**1.000**	0.219
**c5**	0.248	0.237	0.223	0.219	**0.402**

Table S6. Average Pearson correlation among SVC maps associated with the 30 root nodes, referred to the solution with 6 clusters. Within-cluster values are reported in the diagonal of the table, while off-diagonal values refer to between-cluster correlation. The highest value in each row (i.e. for each cluster) is highlighted in bold. Cluster numbers go from the top to the bottom of the dendrogram showed in Figure 2.

	**c1**	**c2**	**c3**	**c4**	**c5**	**c6**
**c1**	0.308	**0.332**	0.312	0.290	0.285	0.249
**c2**	0.332	**0.871**	0.284	0.266	0.260	0.269
**c3**	0.312	0.284	**0.825**	0.286	0.280	0.240
**c4**	0.290	0.266	0.286	**0.956**	0.263	0.227
**c5**	0.285	0.260	0.280	0.263	**1.000**	0.223
**c6**	0.249	0.269	0.240	0.227	0.223	**0.402**

Table S7. Average Pearson correlation among SVC maps associated with the 30 root nodes, referred to the solution with 7 clusters. Within-cluster values are reported in the diagonal of the table, while off-diagonal values refer to between-cluster correlation. The highest value in each row (i.e. for each cluster) is highlighted in bold. Cluster numbers go from the top to the bottom of the dendrogram showed in Figure 2.

	c1	c2	c3	c4	c5	c6	c7
**c1**	0.308	**0.332**	0.312	0.290	0.285	0.271	0.248
**c2**	0.332	**0.871**	0.284	0.266	0.260	0.266	0.265
**c3**	0.312	0.284	**0.825**	0.286	0.280	0.266	0.241
**c4**	0.290	0.266	0.286	**0.956**	0.263	0.248	0.230
**c5**	0.285	0.260	0.280	0.263	**1.000**	0.241	0.228
**c6**	0.271	0.266	0.266	0.248	0.241	**0.394**	0.230
**c7**	0.248	0.265	0.241	0.230	0.228	0.230	**0.534**

Table S8. Average Pearson correlation among SVC maps associated with the 30 root nodes, referred to the solution with 8 clusters. Within-cluster values are reported in the diagonal of the table, while off-diagonal values refer to between-cluster correlation. The highest value in each row (i.e. for each cluster) is highlighted in bold. Cluster numbers go from the top to the bottom of the dendrogram showed in Figure 2.

	c1	c2	c3	c4	c5	c6	c7	c8
**c1**	0.308	**0.332**	0.306	0.301	0.296	0.290	0.275	0.251
**c2**	0.332	**0.871**	0.277	0.275	0.271	0.265	0.270	0.268
**c3**	0.306	0.277	**0.921**	0.285	0.285	0.279	0.269	0.251
**c4**	0.301	0.275	0.285	**0.869**	0.282	0.274	0.265	0.250
**c5**	0.296	0.271	0.285	0.282	**0.956**	0.272	0.257	0.238
**c6**	0.290	0.265	0.279	0.274	0.272	**1.000**	0.250	0.236
**c7**	0.275	0.270	0.269	0.265	0.257	0.250	**0.394**	0.237
**c8**	0.251	0.268	0.251	0.250	0.238	0.236	0.237	**0.534**
